# Neuron-Specific Deletion of the Nf2 Tumor Suppressor Impairs Functional Nerve Regeneration

**DOI:** 10.1371/journal.pone.0159718

**Published:** 2016-07-28

**Authors:** Alexander Schulz, Robert Büttner, Andrea Toledo, Stephan L. Baader, Julia von Maltzahn, Andrey Irintchev, Reinhard Bauer, Helen Morrison

**Affiliations:** 1 Leibniz Institute on Aging, Fritz Lipmann Institute, 07745, Jena, Germany; 2 Institute of Anatomy, Anatomy and Cell Biology, University of Bonn, 53115, Bonn, Germany; 3 Department of Otorhinolaryngology, Jena University Hospital, Friedrich Schiller University Jena, 07747, Jena, Germany; 4 Institute of Molecular Cell Biology & Center for Sepsis Control and Care (CSCC), Jena University Hospital, Friedrich Schiller University Jena, 07747, Jena, Germany; University of Szeged, HUNGARY

## Abstract

In contrast to axons of the central nervous system (CNS), axons of the peripheral nervous system (PNS) show better, but still incomplete and often slow regeneration following injury. The tumor suppressor protein merlin, mutated in the hereditary tumor syndrome Neurofibromatosis type 2 (NF2), has recently been shown to have RhoA regulatory functions in PNS neurons—in addition to its well-characterized, growth-inhibitory activity in Schwann cells. Here we report that the conditional knockout of merlin in PNS neurons leads to impaired functional recovery of mice following sciatic nerve crush injury, in a gene-dosage dependent manner. Gross anatomical or electrophysiological alterations of sciatic nerves could not be detected. However, correlating with attenuated RhoA activation due to merlin deletion, ultrastructural analysis of nerve samples indicated enhanced sprouting of axons with reduced caliber size and increased myelination compared to wildtype animals. We conclude that deletion of the tumor suppressor merlin in the neuronal compartment of peripheral nerves results in compromised functional regeneration after injury. This mechanism could explain the clinical observation that NF2 patients suffer from higher incidences of slowly recovering facial nerve paralysis after vestibular schwannoma surgery.

## Introduction

The successful regeneration of damaged nerve cells due to traumatic injuries or neurodegenerative conditions, displays a desirable, if not yet realistic therapeutic goal. While axons of the CNS are mostly incapable of sufficient regeneration, axons of the PNS generally show a remarkable repair response—due to strong intrinsic growth capacity coupled with permissive environmental factors [1]. Nevertheless, nerve regeneration in the PNS is often incomplete, slow and/or non-functional [2]. In recent years, several molecular targets have been identified that stimulate neuron-intrinsic capabilities to either regenerate or overcome myelin-associated inhibition of regeneration. Small GTPases—and in particular RhoA signals—have proven to be key regulators of both neuromorphogenesis during development and regeneration of the nervous system by local cytoskeleton rearrangements [3]. As a matter of fact, the inhibition of activated GTP-loaded RhoA promotes neuroregeneration [4]. Several lines of evidence also conclusively demonstrate RhoA to be a downstream signaling component of NogoA molecules that prevent axon regeneration in the CNS [5]. Similarly, inhibition of RhoA and its downstream kinase ROCK (Rho-associated kinase), has been shown to enhance nerve regeneration in the peripheral nervous system [6]. Thus, activity regulators of small G-proteins such as RhoA present as promising targets for interfering with the regenerative capacity of neurons.

The tumor suppressor protein merlin [7], mutated in the Neurofibromatosis type 2 tumor syndrome (NF2), is an established regulator of small GTPases [8]. A hallmark of NF2 disease is the development of Schwann cell tumors (schwannomas) at the vestibulocochlear nerve, referred to as vestibular schwannomas. However, schwannomas may affect all parts of the peripheral nervous system. To date, effective treatment modalities mainly involve surgical resection of the tumors—often with the risk of iatrogenic damages to adjacent nerve structures. Thus, facial nerve paralysis, due to the anatomical proximity of the two cranial nerves, is a major side effect of vestibular schwannoma resection [9]. Although no appropriate systematic studies are currently available, clinical observations suggest that recovery from facial nerve palsy is markedly reduced in NF2 patients when compared to individuals with sporadically occurring vestibular schwannomas (personal communication, Steffen Rosahl). This suggests a relevance of *Nf2* germline mutations—detectable in most NF2 patients—for functional nerve recovery, where one allele of the gene is missing in all cells of the organism.

As such, we recently found that merlin has relevant functions in neuronal cell types [10], apart from its growth-inhibiting and tumor-preventing effect in Schwann cells. More specifically, we highlighted that neuronally expressed merlin impacts on RhoA signaling in axons of the PNS; thereby defining axon structure maintenance through the regulation of neurofilament phosphorylation [11]. Given merlin’s ability to control Rho signals, we hypothesized that loss of merlin may alter the regenerative capacity of PNS nerves. Consequently, we performed sciatic nerve crush injuries on mice bearing a conditional knockout of merlin in peripheral motor and sensory neurons, using the neurofilament heavy class (NF-H) promoter to drive cre recombinase activity (Nefh-Cre) [12].

## Materials and Methods

### Experimental animals

All mice were handled in accordance with local governmental and institutional animal care regulations. The protocol was approved by the Thüringer Landesamt für Verbraucherschutz (Permit Number 02-051/13). Nf2flox animals (RIKEN BioResource Centre) were used to obtain conditional neuron-specific merlin knockout by crossing with a mouse line that expresses Cre recombinase under the neurofilament heavy class promoter (Nefh-Cre) (The Jackson Laboratory, USA, stock 009102). The efficiency of this knockout strategy has previously been shown [13]. Cre recombinase-specific genotyping was performed using the following primers: 5’-GGG CCA CCG CGG ATA TAA AA-3’ (forward) and 5’-TGC GAA CCT CAT CAC TCG TT-3’ (reverse) [14]. All mice were on a mixed C57BL/6-FVB/N background.

### Sciatic nerve crush injuries

Unilateral injuries of sciatic nerves were accomplished following previously described methods [15]. Briefly, 8–10-week-old mice were anaesthetized using 2% isoflurane in 100% oxygen. Fur was then removed from one hind limb. After an appropriate incision of the skin, the gluteal musculature was separated in order to reveal the right sciatic nerve. Using hemostatic forceps (Ultra Fine Haemostat, #13021–12, tip width 0.6 mm, Fine Science Tools, Germany), the nerve was crushed once by the application of a defined pressure for 20 s. The locking mechanism of the hemostatic forceps with a series of interlocking teeth ensured reproducibility and standardization of crush injury. Finally, both the gluteal musculature and skin incision were sutured using non-absorbable surgical suture material (Polyethylene terephthalate; USP 4/0, #17218113, Catgut GmbH, Germany). Within the first week after surgery, all mice were monitored on a daily basis for post-operative complications such as pain or wound healing deficits. If necessary, nonsteroidal anti-inflammatory drugs were administered systemically. Finally, all animals were sacrificed by neck dislocation before tissue dissection.

### Single-frame motion analysis (SFMA)

To evaluate locomotor function, mice were accustomed to beam-walking (length 1 m; width 4 cm) one week prior to surgery. For all mice, a rear view of one walking trial was captured with a video camera prior to surgery and at different time-points following surgery. These video sequences were examined using VirtualDub 1.6.19 software. Selected frames, in which the animals could be seen in defined phases of the step cycle, were used for measurements of the foot-base angle (FBA) and lateral foot-base angle (LFBA), as described in [16].

### Mechanical threshold estimation

Sensory regeneration in mice was measured using Semmes-Weinstein filaments, according to previously described methods [17]. Briefly, 5 different monofilaments delivering reproducible forces (#413502, AFH-Webshop, Germany) were applied to stimulate hind paw withdrawal at the mid-plantar region. Scoring was performed as follows: 0 –no response; 1 –response upon 300.0 g pressure; 2–4.0 g; 3–2.0 g; 4–0.4 g; 5–0.07 g.

### Electrophysiology

Investigation of sciatic nerve conduction characteristics was performed 5 weeks after experimental crush injury, using previously described methods [18]. Mice were anesthetized using isoflurane/O_2_ inhalation. Hind limb fur was removed. A constant body temperature of 37°C was maintained by a heating pad and continuously monitored by a rectal thermo probe. Monopolar disposable 28 G needle electrodes were injected at defined positions along the sciatic nerve, in order to perform both proximal and distal nerve stimulation. Needle electrodes were applied because they allow for direct and specific stimulation of the sciatic nerve, without affecting surrounding structures. A ring electrode, superficially installed at the position where the gastrocnemius muscle has its maximum diameter, recorded the neuromuscular response.

### Immunohistochemistry

Paraffin–embedded sciatic nerve sections were rehydrated, boiled in 10 mM sodium citrate buffer (pH9) for 30 min in a microwave and subsequently treated with 0,5% Triton X–100 for 10 min. Sections were incubated for 2 h in 0.2% gelatine and 2% goat serum diluted in PBS. They were submersed in the primary antibody solution overnight at 4°C. Antibodies used: anti-neurofilament (NF; 1:200; BioLegend; SMI312), anti-myelin protein zero (MPZ; 1:200, Abcam, ab39375) and anti-myelin basic protein (MBP; 1:500; Millipore; MAB384). After vigorous washing, the sections were incubated at room temperature for 2 h with secondary antibody solution (Alexa488- and Alexa546-conjugated goat anti-mouse and -rabbit antibodies, 1:500 in PBS, Invitrogen) and counterstained using DAPI (1μg/ml PBS, 10 min).

### NMJ analysis

Extensor digitorum longus (EDL) muscles were fixed in 2% PFA for 15 min, permeabilized with 0.2% Triton X-100 in PBS and then stained with Bungarotoxin-555 (1:500; Invitrogen) and antibodies directed to neurofilaments and SV2 (Developmental hybridoma bank, undiluted).

Immunofluorescence and histological analyses of tibialis anterior (TA) muscles were performed as described previously [19, 20]. For immunofluorescence analyses, an antibody directed against laminin (1:1000; Abcam; ab11575) was used. For occupancy counts, the occupancy of individual neuromuscular junctions was analyzed by categorizing endplates as either fully occupied (neurofilament entirely overlies endplate), partially occupied (neurofilament partially covers endplate) or vacant (no neurofilament overlies endplate).

### Measurement of minimal fiber feret

Cross sections of muscles were stained with antibodies directed against laminin (1:1000, Sigma L9393;). Images were obtained using the Axio Observer Z1 from Zeiss. The minimal fiber feret is defined as closest possible distance between the two parallel tangents of an object, in our case the muscle fiber [21]. The Zeiss Zen software was used to measure the minimal fiber feret.

### Immunoblotting

Immunoblotting was performed as described previously [22]. The following primary antibodies were used: anti-merlin (1:500, Santa Cruz Biotechnology, clone A-19), anti-actin (1:2,000, Santa Cruz Biotechnology, clone I-19), anti-RhoA (1:500, Upstate).

### Active RhoA pull-down

A detection kit was used (Active Rho Pull-Down and Detection Kit, Pierce Biotechnology) in accordance with manufacturer’s instructions. Precipitates and total pooled lysates from three sciatic nerves per genotype were resolved on a 10% SDS-PAGE gel and immunoblotted using an antibody raised against RhoA (1:500, Upstate). Total lysates were used as loading controls.

### Speed of axonal growth in vivo

The right sciatic nerve was dissected 7 days after nerve crush injury and fixed in 4% paraformaldehyde at 4°C for 24 hours. Paraffin–embedded nerves were subsequently used to produce 5- μm-thick serial sciatic nerve cross sections. The automated sectioning mode of the microtome was applied, to collect around 20 sections per millimeter.

### Morphometric analysis of mouse sciatic nerves

Analysis of axon caliber, myelination thickness and axon density, was conducted on sciatic nerve semithin sections removed from transcardially perfused mice. The perfusion solution contained 3% paraformaldehyde and 3% glutaraldehyde in 0.1 M phosphate buffer (pH 7.4). Sections were postfixed for 1 h and kept in a fixative which included 3% sucrose. Images of toluidine blue-stained semithin sections were taken using an Axioskop 2 MOT (Carl Zeiss, Germany), equipped with a 100× immersion oil objective and an Olympus XC50 camera (Olympus, Germany). Standardized settings for camera sensitivity, resolution (2,576×1,932 pixels) and illumination were used for all micrographs. Image analysis was conducted with ImageJ version 1.43u. Employing freehand selection tool, axon and myelin were grossly circumscribed and the area adapted using the ABSnake plugin (the gradient threshold varied between 20 and 30, 10 to 20 iterations were used per image). Based on the areas measured, the thicknesses of the axons and myelin sheaths were calculated. Axons with distorted myelin sheaths were excluded from measurement as fixation artefacts. Axon density was only quantified with regard to myelinated axons.

### Statistical analysis

Comparisons between groups were conducted using an unpaired t test, with one-way or two-way analysis of variance, if appropriate (SPSS software, Statistical Package for the Social Sciences, USA). In case of repeated measurements, two-way analysis of variance with repeated measures was used. Post hoc comparisons were made with the Holm–Sidak test. Differences were considered significant when P < 0.05. All values are presented as means with the corresponding standard errors.

## Results

### Conditional loss of merlin impairs functional motor recovery

**Fig 1:**
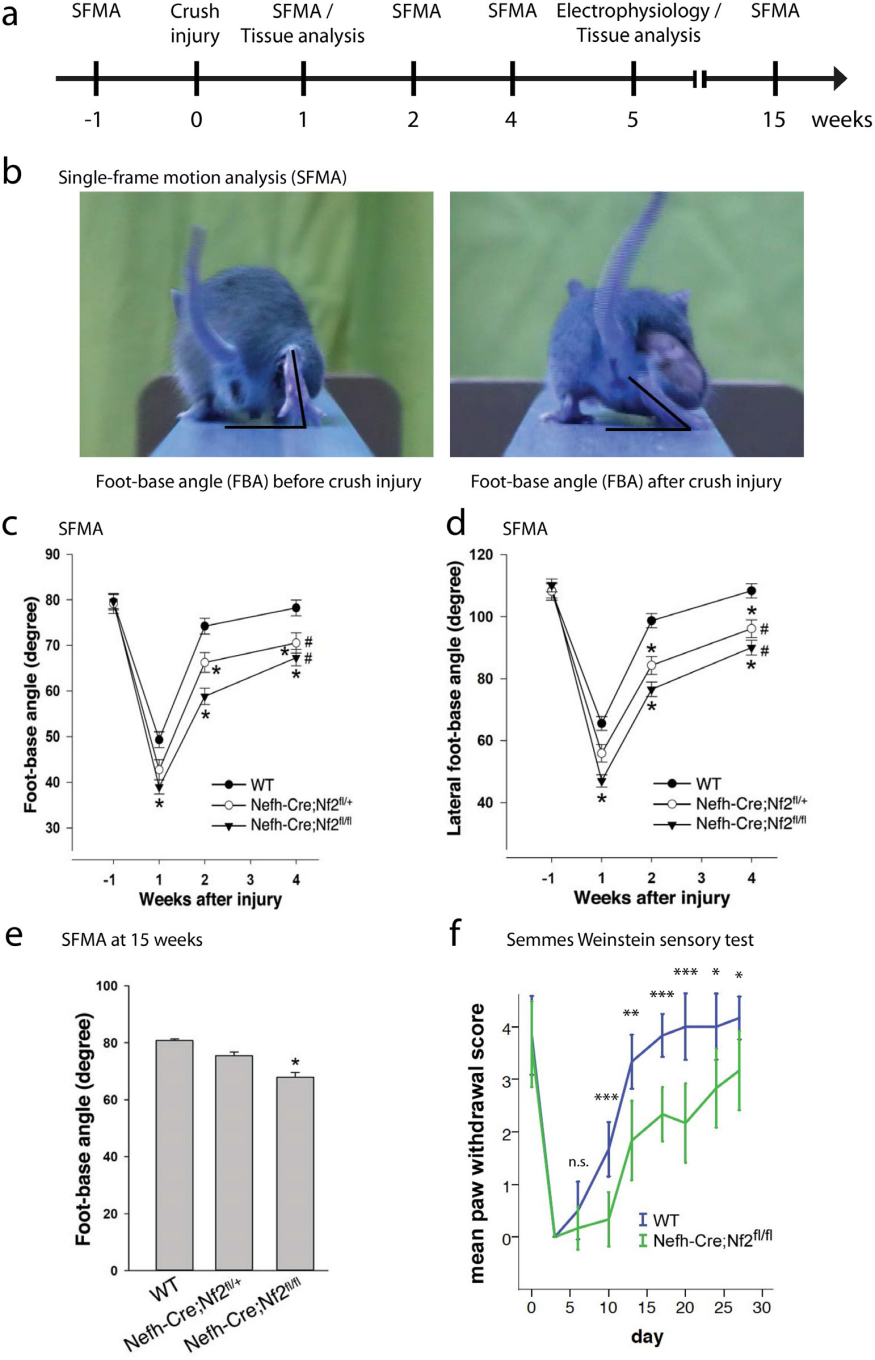
Functional motor recovery following sciatic nerve crush injury. a, Schematic representation of the study protocol. b, Representative images showing SFMA data acquisition. Foot-base angle (FBA) of right hind limb is depicted before (left picture) and after (right picture) sciatic nerve crush injury. c,d, FBA (c) and lateral foot-base angle (LFBA, d) quantification of heterozygous (Nefh-Cre;Nf2^fl/+^) and homozygous (Nefh-Cre;Nf2^fl/fl^) merlin knockout animals following sciatic nerve crush, compared to wildtype (WT) mice. FBA baseline levels were measured before nerve injury (week -1). Functional motor recovery after sciatic nerve crush was assessed for 4 consecutive weeks (* P < 0.05 for differences from WT; # P < 0.05 for differences from baseline within same genotype; two-way ANOVA for repeated measures with Holm-Sidak post-hoc test; n = 8 animals per genotype; mean ± SEM). e, FBA quantification of indicated genotypes 15 weeks after crush injury (*P < 0.05 for differences from WT; one-way ANOVA with Holm-Sidak post-hoc test; n = 8 animals per genotype; mean ± SEM). f, Quantification of paw withdrawal scoring using Semmes Weinstein monofilaments (*P < 0.05; **P < 0.01; ***P < 0.001; n.s. non-significant; n = 6 animals per genotype; mean ± SEM).

In order to study the role of the tumor suppressor protein merlin in peripheral nerve regeneration *in vivo*, mice with a cell type-specific loss of merlin in neurons underwent a defined study protocol (Fig 1a). Single-frame motion analysis (SFMA) was conducted for quantification of functional motor recovery after sciatic nerve crush injury [23]. SFMA sensitively measures the ability of foot extension in animals—a task exclusively dependent on the sciatic nerve—as readout for accurate nerve functionality after crush injury [16]. Therefore, we determined the foot-base angle (FBA) as an indicator of functional motor recovery (Fig 1b).

By FBA monitoring, wildtype animals (WT) show complete functional recovery within 4 weeks of experimental nerve trauma (Fig 1c). The conditional heterozygous and homozygous knockout of merlin in neurons, however, resulted in a significant decline in nerve regeneration—indicated by constantly smaller FBA values over the 4 weeks after crush injury—compared to wildtype littermates (Fig 1c). Measurements of another parameter, the lateral foot-base angle (LFBA) [16], produced consistent results (Fig 1d). Further, when we tested the mice for long-term regeneration, 15 weeks after the experimental injury, only animals with a homozygous loss of merlin in neurons (Nefh-Cre;Nf2^fl/fl^) failed to reach the baseline levels; indicating a durable regeneration deficit. In contrast, animals with heterozygous deletion of merlin in neurons (Nefh-Cre;Nf2^fl/+^) displayed motor recovery comparable to wildtype mice, after 15 weeks (Fig 1e).

Apart from motor neurons of the PNS, the high molecular weight neurofilament subunit (NF-H) has also been consistently shown to be expressed in large dorsal root ganglion (DRG) neurons, but not in small and medium diameter DRG neurons conveying nociceptive stimuli [12, 24–27]. We therefore reasoned that the Nefh-Cre driver line would also delete merlin in sensory fibers of the sciatic nerve responsible for tactile sensations. Indeed, testing of mechanical sensitivity using Semmes-Weinstein monofilaments revealed a significantly delayed recovery of sensory nerve function in conditional *nf2*-deficient mice, following sciatic nerve crush (Fig 1f).

### No electrophysiological alterations in merlin-deficient mice after injury

Electrophysiological measurements are routinely used to investigate functional integrity of peripheral nerves. While the nerve conduction velocity (NCV) measures myelination-dependent rapid signal propagation, the compound motor action potential (CMAP) correlates with the number of functionally recruited motor units by a stimulated nerve [28].

**Fig 2:**
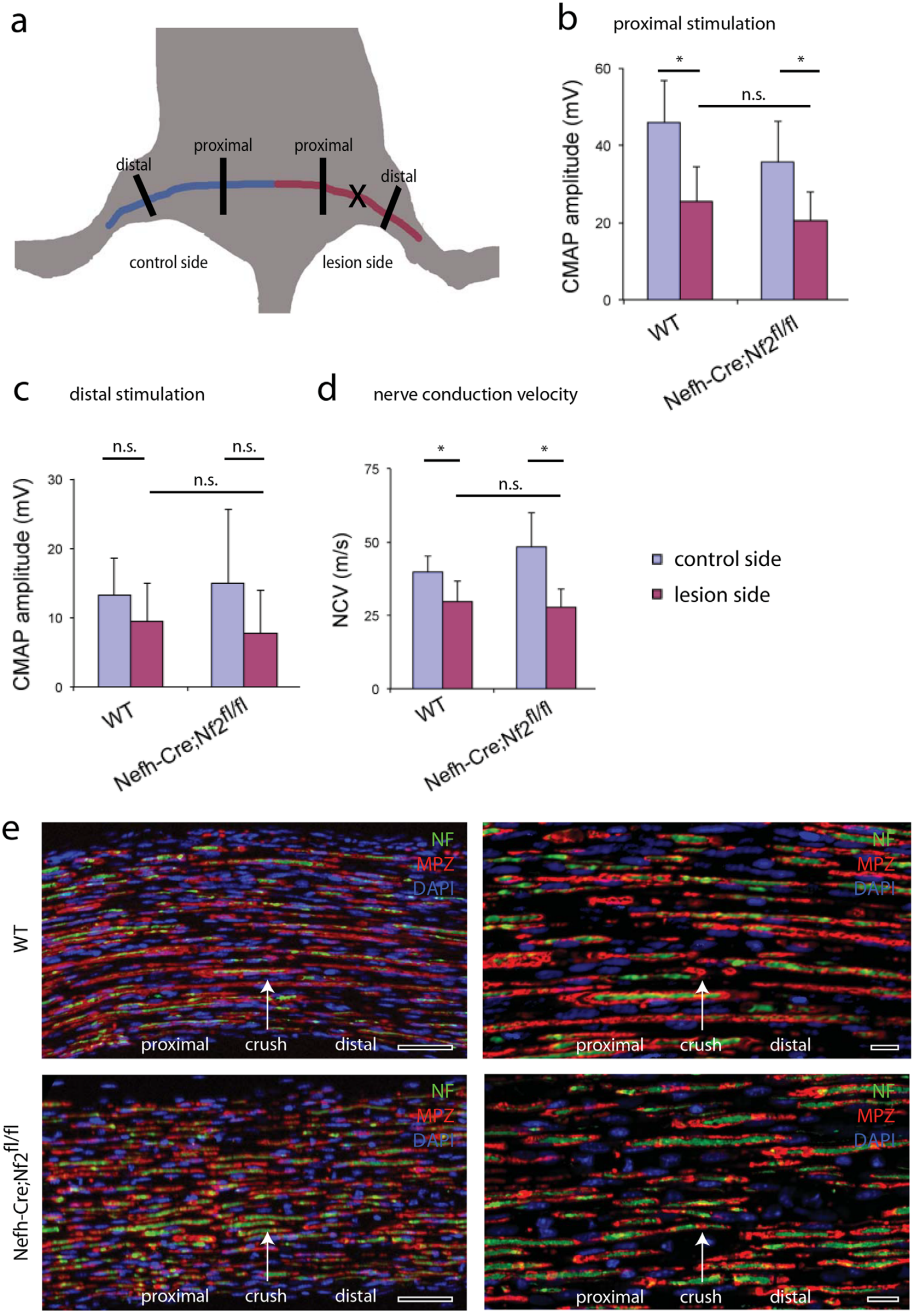
No electrophysiological changes in merlin-deficient mice. a, Schematic drawing of sciatic nerve anatomy *in situ* (right sciatic nerve in red; left sciatic nerve in blue). Cross indicates position of nerve injury in relation to defined proximal and distal positions for electrophysiological measurements and ultrastructural analysis. b-d, Compound motor action potential (CMAP) measurement following proximal (b) and distal (c) stimulation, as well as measurement of nerve conduction velocity, (d) on crushed (lesion side) and non-crushed (control side) sciatic nerves. Genotypes as indicated (*P < 0.05; n.s. non-significant; n = 6 animals per genotype; mean ± SEM). e, Immunohistochemical stainings on longitudinal sections of sciatic nerves taken from wildtype and Nefh-Cre;Nf2^fl/fl^ mice (n = 3) 5 weeks after crush injury. MPZ (myelin protein zero) staining indicates differentiated Schwann cells and myelination, while neurofilament immunolabeling (NF) shows axonal fibers. The arrow shows the position of nerve crush. Orientation of nerves is stated as ‘distal’ and ‘proximal’. Scale bar is 100 μm in the left panel and 20 μm in the right panel.

Following stimulation of the injured sciatic nerve proximal to the crush site, 5 weeks after experimental injury (Fig 2a), CMAP amplitudes are significantly reduced in both wildtype and Nefh-Cre;Nf2^fl/fl^ mice compared to non-injured control nerves (Fig 2b). Genotype-dependent differences, however, could not be observed (Fig 2b). Upon sciatic nerve stimulation distal to the crush site, statistically similar amplitudes were detected for injured and intact control nerves, as well as nerves for wildtype and knockout mice (Fig 2c). The nerve conduction velocity was significantly reduced after crush injury, but was not affected by the neuron-specific loss of merlin (Fig 2d). Furthermore, immunohistochemical stainings of longitudinal sciatic nerve sections, 5 weeks after injury, displayed normal gross morphological features with respect to Schwann cell differentiation, myelination and axonal integrity in knockout animals (Fig 2e and S1 Fig). Taken together, electrophysiological nerve properties are, as expected, affected by nerve crush injury itself. However, neuronal merlin deletion did not intensify the existing CMAP reduction seen in wildtype controls.

**Fig 3:**
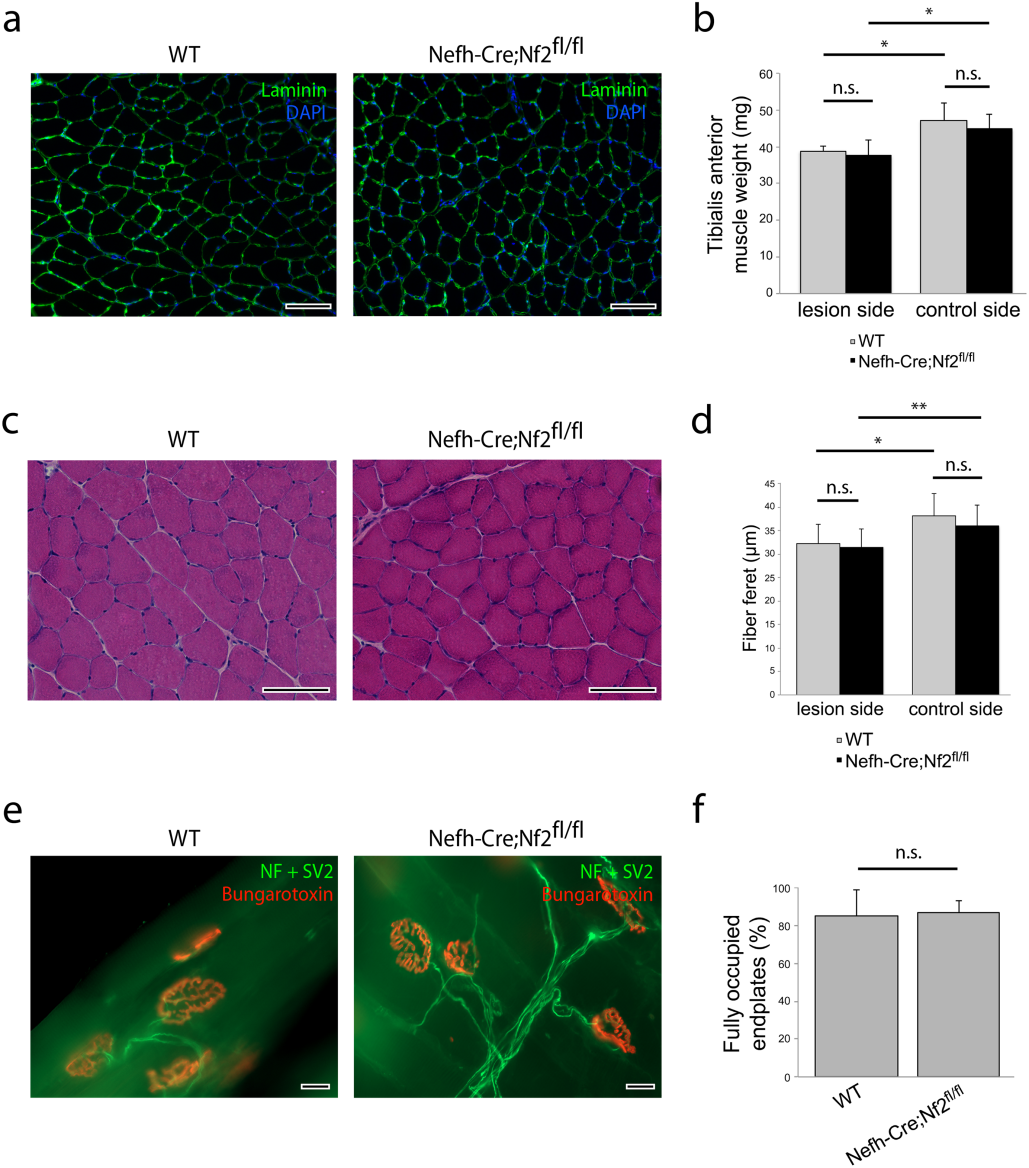
No obvious changes in skeletal muscle in merlin-deficient mice. a, Immunohistochemical stainings of denervated tibialis anterior (TA) muscle sections from wildtype and Nefh-Cre;Nf2^fl/fl^ mice (n = 3), 5 weeks after crush injury. Laminin staining delineates single muscle fibers. Scale bar 100 μm. b, Quantification of tibialis anterior muscle weight from indicated genotypes (*P < 0.05; n.s. non-significant; n = 3 animals per genotype; mean ± SEM). c, Representative photographs of HE stained tibialis anterior muscle fibers. 5 weeks after sciatic nerve crush injury, ipsilateral (lesion side) and contralateral (control side) muscle tissue was prepared from wildtype (WT) and knockout (Nefh-Cre;Nf2^fl/fl^) animals. Scale bar 100 μm. d, Quantification of the minimal fiber feret of tibialis anterior muscles from indicated genotypes was analyzed (*P < 0.05; **P < 0.01; n.s. non-significant; n = 4 animals per genotype; mean ± SEM). e, Representative images of neuromuscular junctions from extensor digitorum longus (EDL) muscle stained with Bungarotoxin (binds to nicotinic acetylcholine receptor) and neurofilaments and SV2. Scale bar 20 μm. f, Quantification of fully occupied endplates in extensor digitorum longus (EDL) muscles from indicated genotypes (n.s. non-significant; n = 3 animals per genotype; mean ± SEM).

Analysis of tibialis anterior (TA) muscles tissue 5 weeks after nerve crush, revealed a regular fine architecture of muscle fibers in merlin knockout animals compared to wildtype littermates (Fig 3a). Muscle weight after crush (lesion side) was not affected by merlin deletion in neurons (Fig 3b). Consistently, the muscle fiber feret reflecting the size of single muscle fibers was decreased after crush injury, but appeared independent of loss of merlin in neurons (Fig 3c and 3d). In addition, assessment of the neuromuscular junctions (NMJ) of extensor digitorum longus (EDL) muscles, indicates a proper re-innervation of muscles following crush injury in Nefh-Cre;Nf2^fl/fl^ animals and control littermates (Fig 3f and 3g).

Hence, both muscle fiber analysis and electrophysiological measurements contradict our data on functional regeneration, which indicated both a delayed motor and sensory recovery.

### Morphometric analysis of sciatic nerve sections after crush

**Fig 4:**
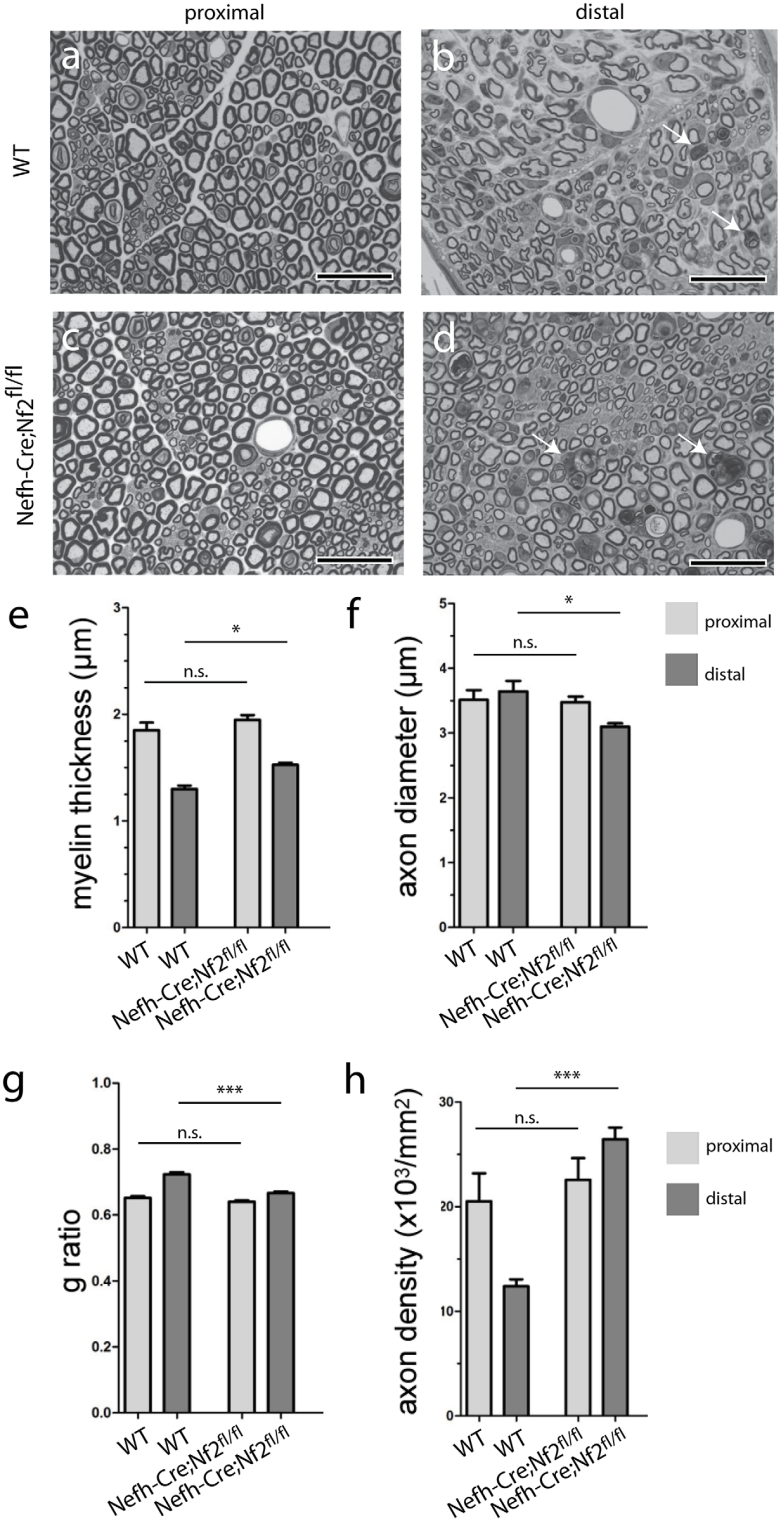
Ultrastructural analysis of sciatic nerves following nerve injury. a-d, Representative images from semithin sciatic nerve cross sections taken from distal and proximal nerve parts, 5 weeks after crush injury. Genotypes as indicated. Arrows indicate degenerating myelin figures. Scale bar 20 μm. e, Quantification of myelin thickness in proximal and distal sciatic nerve sections, 5 weeks after crush injury (*P < 0.05; n = 3 nerves per genotype; mean ± SEM). f, Axon diameter quantification in proximal and distal sciatic nerve sections, 5 weeks after crush injury (*P < 0.05; n = 3 nerves per genotype; mean ± SEM). g, G-ratio quantification in proximal and distal sciatic nerve sections 5 weeks after crush injury (***P < 0.001, n = 3 nerves per genotype; mean ± SEM). h, Quantification of axon density as number of myelinated axons per area in proximal and distal sciatic nerve sections, 5 weeks after crush injury (***P < 0.001; n = 3 nerves per genotype; mean ± SEM).

In order to examine the structural composition of crushed nerves, we analyzed nerve specimens 5 weeks after injury using semithin sections of proximal and distal nerve parts. Wildtype animals showed signs of mild structural degeneration in nerve parts distal to the lesion site (Fig 4b), in comparison to proximal parts (Fig 4a). Distal nerve sections of Nefh-Cre;Nf2^fl/fl^ mice, however, contain axons with smaller diameters and more collagen pockets as indicators for denervated Schwann cell processes (Fig 4d), compared to sections proximal to the injury site (Fig 4c). Statistical analysis of nerve parameters revealed significantly increased myelination thickness (Fig 4e), but also a reduced average axon diameter (Fig 4f) in the distal nerve part of Nefh-Cre;Nf2^fl/fl^ mice in comparison to wildtype littermates. Merlin deficiency in the neuronal compartment of PNS axons consequently results in a slight g-ratio decline, which is a parameter that mathematically integrates myelin thickness and axon diameter (Fig 4g). Strikingly, the total number of myelinated sciatic nerve axons distal to the crush site showed a 2-fold increase in Nefh-Cre;Nf2^fl/fl^ mice compared to wildtype littermates (Fig 4h). The latter finding not only suggests enhanced axonal sprouting in nerves of Nefh-Cre;Nf2^fl/fl^ mice compared to wildtype animals; it further implies that full functional nerve regeneration in wildtype animals (Fig 1c and 1f) can occur, despite reduced axonal numbers in the nerve distal to crush injury compared to the proximal part (Fig 4h).

### Axonal outgrowth in early regeneration

**Fig 5:**
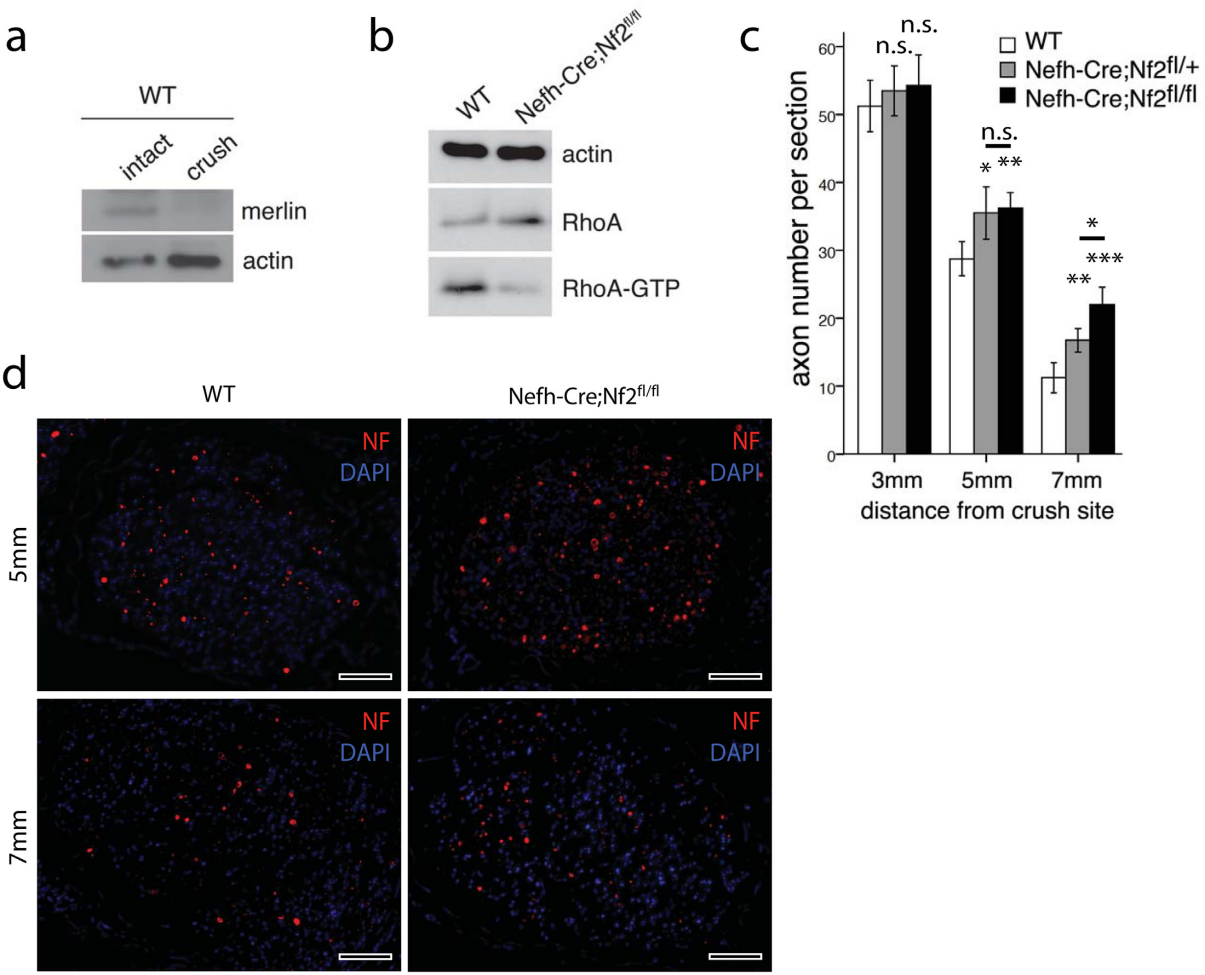
Enhanced axon sprouting in merlin knockout mice. a, Western blot of pooled sciatic nerve lysates from at least 3 wildtype animals. Tissue was collected 7 days after crush injury (crush), as well as from intraindividual non-crushed controls (intact). Immunoblotting for merlin and actin as loading control (n = 3). b, Western blot of pooled sciatic nerve lysates taken from wildtype (Nefh-Cre;Nf2^+/+^) and merlin knockout animals (Nefh-Cre;Nf2^fl/fl^). Active RhoA (RhoA-GTP) is visualized after RhoA pull-down. Actin and RhoA serve as loading control (n = 3). c, Quantification of NF-positive axons per cross section at 3, 5 and 7 mm distal to the crush, 7 days after injury density in distal sciatic nerve sections, 5 weeks after crush injury. Genotypes as indicated (*P < 0.05; **P < 0.01; ***P < 0.001; n.s. non-significant; n = 3 nerves per genotype; mean ± SEM). d, Representative image of sciatic nerve cross section 7 days after crush injury, 5 and 7 mm distal to the crush position. Immunolabeling of neurofilaments (NF) as axon marker and DAPI counterstaining. Scale bar 50 μm.

In order to achieve mechanistic insights into the increased number of axons in Nefh-Cre;Nf2^fl/fl^ mice after crush injury, we further investigated nerve regeneration 7 days after injury—the time point at which axon outgrowth reaches its peak [29]. Interestingly, in sciatic nerve lysates from wildtype mice, merlin protein was markedly down-regulated one week after nerve crush (Fig 5a). Furthermore, activity of RhoA was found to be relevantly decreased following neuron-specific knockout of merlin, as indicated by lower GTP loading one week after crush (Fig 5b). This is in line with our previous reports on the regulation of the molecular switch RhoA by merlin [11]. As RhoA inhibition has been shown to promote neurite extension and axon regeneration, we analyzed the number of regenerating axons in sciatic nerve cross sections 7 days after crush injury (Fig 5c). While the number of neurofilament-positive axons was similar in all tested genotypes 3mm distal to the crush site, the number of axons 5mm,–and even more prominently 7mm, distal to the injury site was significantly higher in mice with heterozygous and homozygous merlin deletion in neurons (Fig 5d)—indicating increased axon sprouting after injury in animals with merlin deficiency.

## Discussion

Mutations of the tumor suppressor protein merlin are found in virtually all sporadic schwannomas, as well as—when affecting the germline—the hereditary tumor syndrome Neurofibromatosis type 2 (NF2). Although NF2 is predominantly characterized by bilateral vestibular schwannomas, affected individuals may suffer from meningeomas, ependymomas, peripheral neuropathy and lesions affecting skin and eyes. The complexity of NF2 disease reflects the importance of the *Nf2* gene encoded protein merlin in various cell types of the human body. Apart from its well-characterized function in Schwann cells [30], we recently reported on neuron-intrinsic functions of merlin [10, 11]. However, the role of merlin in neurons and the relevance of neuronally expressed merlin for NF2 disease, merits more detailed exploration [31].

Concordantly, we investigated the relevance of neuronal merlin for peripheral nerve regeneration in an injury model. By means of sciatic nerve crush injuries on conditional knockout animals, we found a significant decline in functional recovery of motor and sensory sciatic nerve fibers. However, the presented data on compound motor action potentials (CMAP), as well as on re-innervation of muscular endplates, suggests that neuromuscular transmission *per se* is unaffected by the neuron-specific loss of merlin. Sprouting axons after nerve crush injury seem to re-innervate their appropriate targets in merlin deficient mice.

The discrepancy between compromised functional nerve recovery and the ability of re-growing axons to indeed reach their targets, further implies the existence of other confounding factors for comprehensive nerve repair than solely re-innervation of target structures. This inconsistency between functional repair and electrophysiological / NMJ findings could be attributed to ultrastructural nerve alterations. Indeed, distal sciatic nerve sections of mice with conditional *nf2* deletion show twice as many axons with significantly reduced diameters following crush injury. Furthermore, loss of merlin in neurons also impacts on myelination thickness, reinforcing the concept that merlin in the neuronal compartment has functional consequences for adjacent Schwann cells [13]. The unusual combination of smaller-sized axons bearing more elaborate myelin sheaths in merlin mutants, may further explain nerve conduction properties that are comparable to wildtype mice; although these ultrastructural nerve alterations are not sufficient to compensate for the delay in functional nerve regeneration.

Interestingly, our data on the nerve regeneration of wildtype mice suggests that full functional nerve repair occurs despite a significantly reduced number of myelinated axons in the nerve distal to the crush site (Fig 4h). In order to properly address the complexity of peripheral nerve regeneration, other potential, so far unidentified, parameters beyond axonal re-growth should therefore be taken into consideration.

In the absence of neuronal merlin, early axonal outgrowth after nerve crush is seemingly enhanced, which correlates with reduced levels of active RhoA in sciatic nerve lysates. A comparable phenotype, displaying small axon calibers in combination with abundant axonal sprouting, was described in mice bearing a knockout in the neurofilament light (NF-L) protein [32], interpreted as delayed maturation of regenerating fibers.

Functional regeneration of nerves is assumed to require both axonal regrowth and remyelination of axons by Schwann cells. However, our study indicates that both essential steps do not necessarily match with functional recovery *in vivo*. Studies with a primary focus on axon outgrowth as readout for improved nerve regeneration, might therefore overlook the clinically relevant functional component of regeneration—which might be also influenced by other as yet unidentified factors. Based on our results, we have to assume that stimulating signals need to be transmitted to the end organs, along the nerve, in a highly precise and coordinated fashion. Even minor alterations in the ultrastructure of nerves may disturb the sensitive and complex events necessary for functional regeneration.

Conclusively, we provide experimental evidence that the *nf2* gene encoded protein merlin impacts on nerve recovery capacity, underlining the recently discovered importance of merlin in PNS neurons [31]. Furthermore, these findings potentially explain the clinical observations that NF2 patients carrying *Nf2* germline mutations, show poor recovery from postoperative facial nerve palsy, in comparison to individuals with sporadically occurring vestibular schwannomas.

## Conclusions

The tumor suppressor protein merlin is mutated in the most frequent hereditary tumor syndrome of the central and peripheral nervous system—Neurofibromatosis type 2 (NF2). While its tumor-restricting role in glial cell types is undeniable, evidence for functionality of merlin in neurons is still emerging. Here we show that the cell type-specific loss of merlin in neurons, leads to impaired peripheral nerve regeneration following sciatic nerve crush injury *in vivo*. These findings may explain why NF2 patients show poor recovery from postoperative facial nerve palsy, in comparison to individuals with sporadically occurring vestibular schwannomas.

## Supporting Information

S1 Fig Immunohistochemical stainings on longitudinal sections of sciatic nerves taken from wildtype and Nefh-Cre;Nf2^fl/fl^ mice (n = 3), 5 weeks after crush injury. MBP (myelin basic protein) staining indicates myelination. Arrow in each picture shows position of nerve crush. Orientation of nerves is stated as ‘distal’ and ‘proximal’. Scale bar 50 μm.
(EPS)
